# Transcriptomics Reveals the Differences in mRNA Expression Patterns in Yak Uterus of Follicular, Luteal, and Pregnant Phases

**DOI:** 10.3390/ani15060837

**Published:** 2025-03-14

**Authors:** Shaohui Beng, Daoliang Lan, Yueyue Li, Deping Li, Yuehuan Zhang, Zelang Ma, Jianbo Zhu, Shunyang Liu, Kechao Chen, Jian Li, Peng Wang, Wei Fu

**Affiliations:** 1 Key Laboratory of Qinghai-Tibetan Plateau Animal Genetic Resource Reservation and Utilization of Ministry of Education, Southwest Minzu University, Chengdu 610041, China; bangshaohui666@163.com (S.B.); landaoliang@163.com (D.L.); liyueyue0214@163.com (Y.L.); liushunyang2023@163.com (S.L.); chenkechao0612@163.com (K.C.); lijian@swun.edu.cn (J.L.); 2 Sichuan Ganzi Tibetan Autonomous Prefecture, Institute of Animal Husbandry Science, Kangding 626000, China; 7410ldp@sina.com (D.L.); zyh13551981882@163.com (Y.Z.); zeliangm36@163.com (Z.M.); 17381703626@139.com (J.Z.); 3 Key Laboratory of Animal Science of National Ethnic Affairs Commission of China, Southwest Minzu University, Chengdu 610041, China

**Keywords:** yak, transcriptomics, follicular phase, luteal phase, pregnant phase

## Abstract

The yak, an important livestock mostly living in the Qinghai–Tibet Plateau region, has attracted massive attention due to its reproductive specificity. However, the molecular mechanism regulating yak uterine functions remains to be explored. This study utilized transcriptomics to identify differentially expressed genes (DEGs) in yaks across uteruses undergoing the follicular phase (UFP) (*n* = 3/group), luteal phase (ULP) (*n* = 3/group), and pregnant phase (UPP) (*n* = 3/group), aiming to depict and compare their transcriptomic characteristics. For the UFP and ULP groups, 495 DEGs were identified, including 329 upregulated and 166 downregulated DEGs in group ULP compared to UFP. Advanced analysis indicated that these DEGs between UFP and ULP were primary participants in GO items, such as adhesion, cell differentiation, and development, and were significantly enriched in KEGG signaling pathways like arachidonic acid metabolism, retinol metabolism, and cAMP signaling pathways. For the UFP and UPP groups, 353 DEGs were obtained, comprising 198 upregulated and 155 downregulated DEGs in group UPP compared to UFP. Advanced analysis showed that these DEGs between UFP and UPP were mainly related to GO items such as adhesion, binding, and the extracellular region, and were prominently enriched in KEGG signaling pathways like ECM-receptor interaction, the relaxing signaling pathway, and focal adhesion. For the groups ULP and UPP, 1303 DEGs were identified, encompassing 709 upregulated and 594 downregulated DEGs in group UPP compared to ULP. Advanced analysis indicated that these DEGs between ULP and UPP were associated with GO items such as multicellular organismal processes, cell differentiation, and the extracellular region, and mainly gathered in KEGG signaling pathways like signal transduction, cell differentiation, metabolism, and autophagy. These results provide valuable insights into the key biomarkers observable via dynamic changes in the yak uterus and offer a theoretical basis for further studies on yak reproductive mechanisms and improving production performance.

## Simple Summary

Yaks are an important resource for the daily life of herders living in the Qinghai–Tibet Plateau. This study used RNA-seq technology to compare the mRNA expression patterns of the yak uterus during the follicular phase, the luteal phase, and the pregnant phase (*n* = 3/group). Numerous differentially expressed genes (DEGs) were obtained, and further enrichment analysis revealed that these DEGs were mainly associated with metabolism, adhesion, embryo implantation, and fetal growth and development. This study investigates the regulatory mechanisms of the yak uterus among different reproductive phases, providing theoretical references for further research on yak reproductive characteristics.

## 1. Introduction

The yak (*Bos grunniens*) is a special livestock animal that inhabits the high-altitude regions of the Himalayas and its surrounding areas at elevations over 3000 m [1], which predominantly contributes to the development of the local economy and society [2]. Yaks are mainly distributed in China, Mongolia, India, Kazakhstan, Russia, and Nepal [3], with over 95% of the population existing in the southwest and northwest of China, including Xizang, Sichuan, Qinghai, Xinjiang, and Gansu provinces [4]. Yaks are typical seasonal breeders that always exhibit reproductive behaviors such as estrus and mating from June to September each year. Due to their special habitat, yaks mainly have to expend their energy to counteract the harsh environment, which compromises their reproductive capacity. Hence, most female yaks only express a few estrous cycles per breeding season, which can be divided into the follicular phase and luteal phase, with an average duration of 19 to 21 days [5]. Once mating occurs successfully, more than half of the female yaks may become pregnant, and the gestation period is generally around 255 days [6]. Compared to other cattle breeds (e.g., beef cattle, dairy cattle, and buffalo), yaks exhibit a delayed puberty, prolonged sexual maturity, and shorter reproductive lifespans, contributing to the low reproductive efficiency [7,8].

The uterus is the core reproductive organ in mammals and exhibits periodical changes during the estrous cycle [9]. The crucial physiological functions of the uterus mainly include responding to hormone stimuli from the hypothalamic pituitary gonadal (HPG) axis [10], as well as providing the physiological conditions for placenta formation, fetal growth, and development [11]. Similar to other bovine species, the uterus of yaks can be divided into two sections by a longitudinal septum within the uterine horns, classifying it as a bipartite uterus [9]. The tissue structure of yak uteruses mainly consists of three layers from the inside out: the endometrium, myometrium, and perimetrium, with the endometrium being the critical site for embryo implantation [12]. Additionally, a yak uterus not only provides a safe and comfortable environment for fetal growth but also assists in labor through the process of muscle contraction. After delivery, it undergoes a series of complex physiological changes to return to a non-pregnant state, namely, uterine involution [13]. Studies have shown that the ovaries regulate the cyclic changes of the endometrium by secreting hormones (e.g., estrogen and progesterone) [14]. Meanwhile, the uterus can provide feedback regulation to the normal cycle of the ovaries by stimulating the degeneration of the ovarian corpus luteum [15]. Prior to the initiation of fertilization, the cervical mucus secreted by the uterus protects and selects sperm, influencing the success of the fertilization process [16]. After the early embryos enter the uterus from the fallopian tube, the uterus supports their development by inducing embryonic gene expression [17]. The abundant glands and their secretions within the endometrium play a critical role in maintaining normal maternal pregnancy [18]. Simultaneously, the caruncles on the uterine wall fuse with the fetal chorion to form the placenta, a pivotal region facilitating gas and nutrient exchange between the maternal and fetal circulatory systems [19]. The success of bovine pregnancy hinges on the initiation of maternal–embryo interactions during early gestation [20]. The uterus, serving as the primary site for this biological dialogue, assumes a central role in supporting gestation in female livestock.

The uterus undergoes complex structural and physiological changes during the follicular, luteal, and pregnant phases. With the continuous advancement of high-throughput sequencing, RNA-seq technology has gained extensive utilization in genomic research, increasingly becoming an important tool for deciphering the transcriptional regulatory mechanisms behind phenotypes. Recent investigations into the bovine endometrial transcriptome have predominantly elucidated molecular mechanisms underlying embryo–maternal crosstalk [21,22], pregnancy recognition signaling [23], and immunomodulatory pathways [24]. However, there is a notable dearth of research concerning the molecular functions of the yak uterus across various reproductive cycles, underscoring the need for more comprehensive investigations. This study applies RNA-seq technology to investigate the yak uterus across different reproductive cycles, comparing and analyzing the changes of gene expression during the follicular, luteal, and pregnant phases, with the aim of identifying key pathways and genes associated with the function of the yak uterus. Our findings aim to establish a theoretical framework for further understanding the molecular mechanisms of physiological activities in the yak uterus.

## 2. Methods and Materials

### 2.1. Sample Collections

Healthy female yaks aged 4–6 years, raised under consistent conditions, were specifically chosen for this study. Uterine tissue samples representing the follicular phase, luteal phase, and three months of pregnancy (including the chorioallantoic membrane) were collected post-slaughter. These samples were collected within one year, with three samples selected from each stage for RNA-seq analysis. Following collection, the samples were rinsed with physiological saline containing a 1% penicillin–streptomycin mixture (Thermo Fisher Scientific Inc., Waltham, USA) and immediately frozen in liquid nitrogen. All samples were stored in a −80 °C ultra-low temperature freezer (Thermo Fisher Scientific Inc., Waltham, USA) until required for analysis. The animal experiments were checked and approved by the Ethics Committee of Southwest Minzu University (the Ethic Approval code: SMU-202401165) and performed in accordance with the Animal Care and Use Program Guidelines of Sichuan Province, China.

### 2.2. Real-Time Quantitative Polymerase Reaction (RT-qPCR)

According to the cDNA sequence of yak genes provided by GenBank, primer design was carried out through the NCBI website (https://blast.ncbi.nlm.nih.gov/Blast.cgi, accessed on 25 May 2024). The primer sequences are detailed in Table 1, and these primers were synthesized by Beijing Tsingke Biotech Co., Ltd. (Beijing, China). The internal reference gene *β-ACTIN* (NM_173979.3) was employed to assess the expression levels of *GSN* (XM_014476296.1), *VIM* (XM_005891917.2), *FOS* (XM_005900989.2), and *GLI1* (XM_005901692.1) genes in yak uterus. Total RNA was extracted from yak uterine tissues by using Total RNA Extraction Kit (Biosharp, Hefei, China), followed by cDNA synthesis via a two-step method as per the kit’s instructions. The RT-qPCR reaction mixture consisted of 5 μL of SYBR Premix Ex Taq^TM^ II (Vazyme, Nanjing, China), 1 μL of cDNA, 0.5 μL of Forward primer (10 μmol/L), 0.5 μL of Reverse primer (10 μmol/L), and 3.0 μL of Rnase free ddH_2_O. The RT-qPCR reaction was conducted as follows: pre-denaturation at 95 °C for 30 s; denaturation at 95 °C for 10 s, and annealing at 60 °C for 15 s, with 40 cycles of replication. Data analysis for relative quantification was performed using the 2^-△△Ct^ method, and GraphPad Prism10.1.2 (GraphPad Software Company, La Jolla, CA, USA) was utilized for statistical analysis and graphical representation. This study incorporated three biological replicates and three technical replicates to ensure robustness and reliability of the results.

### 2.3. RNA Sequencing

#### 2.3.1. Construction of cDNA Library and Transcriptome Sequencing

The uterine tissue samples of yaks during the follicular, luteal, and pregnancy phases were placed in liquid nitrogen and sent to Shanghai Personal Biotechnology Co., Ltd. (Shanghai, China) for RNA-seq sequencing and subsequent analysis. The samples underwent standard processing procedures: total RNA was extracted using the Trizol reagent kit (Thermo Electron Corporation, US), and RNA integrity, concentration, and purity were assessed through 1% agarose gel electrophoresis and spectrophotometry.

Following quality assessment, mRNA enriched with Oligo (dT) (Thermo Fisher™, Waltham, MA, USA) beads was randomly fragmented. Fragmented mRNA served as a template for cDNA synthesis, which underwent purification and end-repair steps before PCR amplification and purification of the PCR products, ultimately constructing the library. Based on the Illumina™ NovaSeq 6000^®^ sequencing platform from Shanghai Personal Biotechnology Co., Ltd. (Shanghai, China), paired-end (PE) sequencing was performed on the library using Next-Generation Sequencing (NGS) technology.

#### 2.3.2. RNA-Seq and Data Processing

After sequencing, image files were generated and converted using the software provided by the sequencing platform to generate the raw FASTQ (v0.23.4) data. Subsequently, key metrics such as sample names, total base counts, Q30 (pb), percentage of ambiguous bases, Q20 (%), and Q30 (%) were calculated. Data filtering was performed using Cutadapt (v1.9.1) to remove 3’ adapter sequences, eliminating those with a minimum 10 bp overlap with known adapters (AGATCGGAAG), permitting up to 20% base mismatches, and removing reads with an average quality score below Q20 to obtain high-quality, reliable clean reads.

The read count values for the aligned genes were calculated using HTSeq (v2.0.3) to obtain the raw expression levels of the genes. Subsequently, Fragments Per Kilobase per Million fragments (FPKM) was employed to normalize gene expression levels. FPKM represents the number of fragments derived from a specific gene per kilobase of its length per million fragments. A gene is considered expressed when FPKM > 1.

Genes and transcripts with |log_2_(Fold Change) > 1| and FDR < 0.05 were filtered for differentially expressed genes (DEGs) and subjected to biological analysis. The R package Pheatmap (v1.0.12) was used for two-way clustering analysis of the union of DEGs and samples from the follicular phase, luteal phase, and pregnancy phase. Clustering was performed based on the expression levels of the same gene across different samples and the expression patterns of different genes within the same sample. The Euclidean method was employed for distance calculation, and complete linkage was utilized for hierarchical clustering.

#### 2.3.3. Enrichment Analysis of Differentially Expressed Genes (DEGs)

GO enrichment analysis (http://geneontology.org/: accessed on 19 May 2024): GO enrichment analysis was performed using topGO (Release 2.58.0). Differential genes were annotated with GO terms to ascertain the gene list and count per term. The *p*-value, derived via the hypergeometric distribution method, was computed to identify significantly enriched GO terms (*p* < 0.01) among the differential genes. This analysis aimed to unveil the primary biological functions executed by the differential genes.

KEGG enrichment analysis (http://www.kegg.jp/: accessed on 19 May 2024): KEGG enrichment analysis was performed using clusterprofiler (v4.10.0). The gene list and gene count per pathway were determined using the KEGG pathway-annotated differential genes. Subsequently, the *p*-value was calculated by the hypergeometric distribution method, with a significance threshold of *p* < 0.05, to identify significantly enriched KEGG pathways associated with the differential gene set. This analysis aimed to elucidate the primary biological functions governed by the differential genes within the pathways.

## 3. Results

### 3.1. Transcriptome Reveals the Gene Expression Patterns in Yaks Among the Uterus of Follicular Phase (UFP), the Uterus of Luteal Phase (ULP), and the Uterus of Pregnant Phase (UPP)

**Figure 1:**
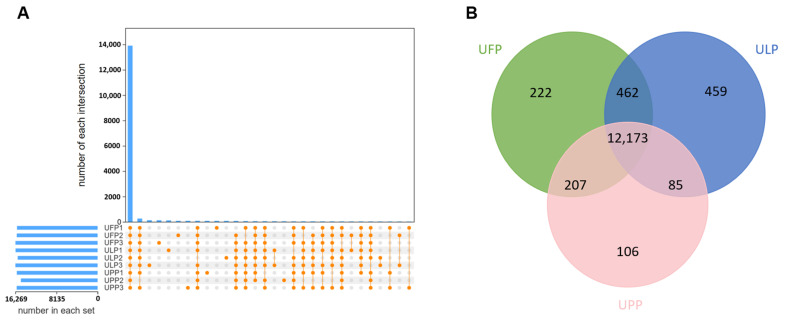
(**A**) Gene expression analysis chart for each sample group. Horizontal bars indicate the total number of genes identified in each sample. Vertical bars represent the number of genes identified as shared between multiple samples, with the line connecting all points representing genes common across all samples. Intersections indicate the number of genes shared between different groups of samples, while unique genes for individual samples are indicated by isolated points. (**B**) Venn diagram of the genes detected in each group. Only genes with FPKM > 1 were counted and displayed in the graph. Green represents the follicular phase uterus, blue represents the luteal phase uterus, and pink represents the pregnant uterus.

Based on the transcriptomic data obtained in the present study, we counted and analyzed the distribution of genes identified across nine samples in the UFP, ULP, and UPP groups. The results showed a substantial gene presence in each sample, with sample ULP3 notably exhibiting the highest count of 16,269 identified genes. Across the nine samples, a comparable number of genes were detected, with 13,904 genes being common to all samples. Additionally, we found that unique genes were identified in individual samples or specific combinations of samples (Figure 1A). Subsequently, genes with an average FPKM > 1 were chosen for additional scrutiny, revealing 12,173 common genes shared among UFP, ULP, and UPP groups. Additionally, 222, 459, and 106 genes were found to be uniquely expressed in the UFP, ULP, and UPP groups, respectively (Figure 1B and Figure S1). Those results indicated a general consistency in the gene expression profiles across the different groups.

**Figure 2:**
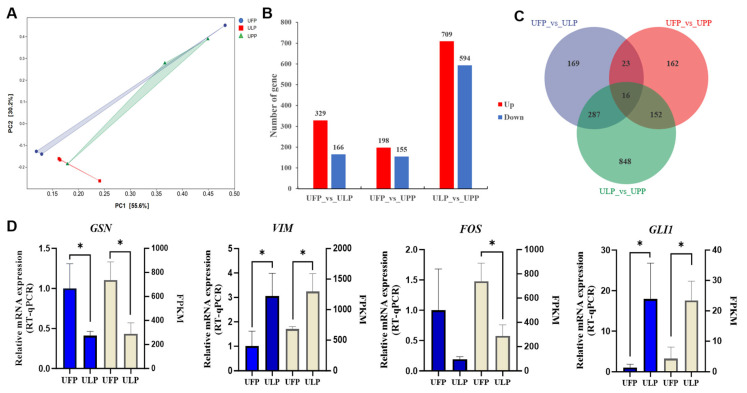
(**A**) Principal components analysis (PCA) of sample distribution in each group. (**B**) Bar chart of differentially expressed genes (DEGs). (**C**) Venn diagram showing the DEGs among the paired comparisons. (**D**) RT-qPCR validation of selected DEGs (*n* = 3); * *p* < 0.05.

Furthermore, we analyzed the differences in gene expression patterns among the groups. Principal components analysis (PCA) showed that PC1 and PC2 accounted for 55.6% and 30.2%, respectively. Additionally, distinct separations were observed among the samples in groups UFP, ULP, and UPP in the PCA plot, indicating significant differences in gene expression profiles among these groups (Figure 2A). In the UFP and ULP group, there were 495 DEGs, with 329 upregulated and 166 downregulated in ULP compared to UFP (Supplementary Table S1). Similarly, in the UFP and UPP group, there were 353 DEGs, with 198 upregulated and 155 downregulated in UPP compared to UFP (Supplementary Table S2). In the ULP and UPP group, there were 1303 DEGs, with 709 upregulated and 594 downregulated in UPP compared to ULP (Figure 2B) (Supplementary Table S3). These DEGs were visualized using a heatmap (Supplementary Figure S1). The Venn diagram results showed that 169, 162, and 848 DEGs were uniquely detected in UFP vs. ULP, UFP vs. UPP, and ULP vs. UPP, respectively, with 16 DEGs shared among the three paired comparisons (Figure 2C). To verify the reliability of the transcriptomic data in this experiment, four highly expressed DEGs were selected, and their mRNA expression was validated using RT-qPCR. The results demonstrated concordance between the qPCR data and the trends observed in the transcriptomic analysis (Figure 2D), underscoring the reproducibility of our experimental findings.

### 3.2. Comparative Analysis of Gene Expression Patterns in Yak Uterus During the Follicular Phase (UFP) and Luteal Phase (ULP)

**Figure 3:**
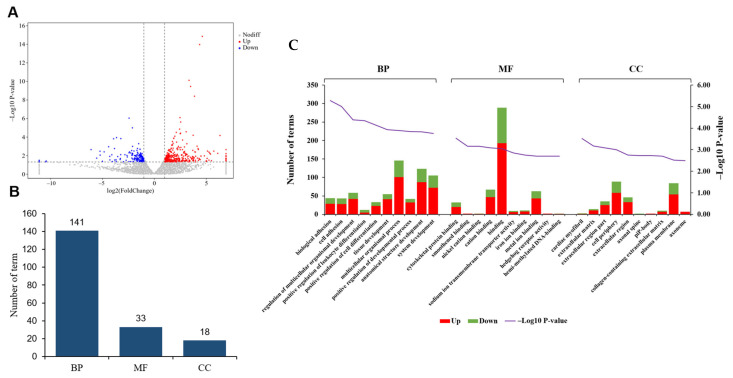
(**A**) The volcano plot of the DEGs between yak uterus during the follicular phase (UFP) and luteal phase (ULP). (**B**) GO terms enriched in biological processes (BP), molecular functions (MF), and cellular components (CC). (**C**) GO classification of DEGs; the left horizontal axis represents the number of enriched genes, while the right vertical axis represents -log_10_ (*p*-value).

Subsequently, we conducted a pairwise analysis of gene expression patterns between the two groups. Initially, we presented the DEGs between UFP and ULP using a volcano plot (Figure 3A). Following this, we performed a comprehensive statistical assessment of the GO classification, revealing that these DEGs were significantly enriched in a total of 192 terms (*p* < 0.01), comprising 141 in biological processes (BP), 33 in molecular functions (MF), and 18 in cellular components (CC) (Figure 3B and Supplementary Table S4). According to the *p*-value, we found that the top three terms enriched in BP were biological adhesion, cell adhesion, and the regulation of multicellular organismal development. In terms of MF, the top three enriched terms were cytoskeletal protein binding, smoothened binding, and nickel cation binding. Furthermore, in the CC category, the top three enriched terms were the cardiac myofibril, extracellular matrix, and extracellular region part (Figure 3C).

**Figure 4:**
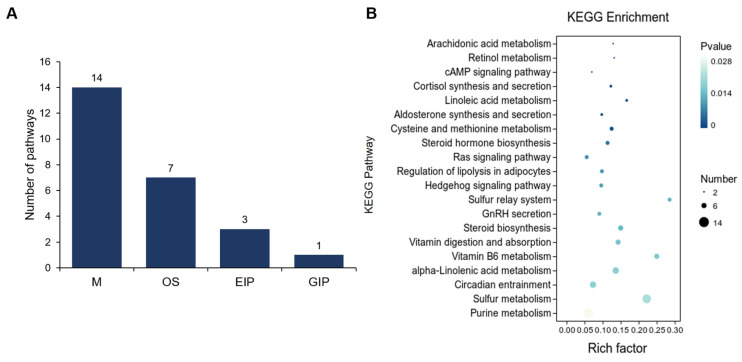
(**A**) Functional classification distribution of KEGG pathway enrichment analysis between yak uterus during the follicular phase (UFP) and luteal phase (ULP). The functional classifications include metabolism (M), organismal systems (OS), environmental information processing (EIP), and genetic information processing (GIP). (**B**) Bubble chart of the top 20 KEGG pathways.

Further, we performed a KEGG enrichment analysis of the DEGs between UFP and ULP, resulting in a total of 25 significant pathways (*p* < 0.05), including 14 pathways enriched in metabolism (M), 7 in organismal systems (OS), 3 in environmental information processing (EIP), and 1 in genetic information processing (GIP) (Figure 4A). We found that the pathways enriched in metabolism mainly include arachidonic acid, retinol, and linoleic acid metabolism. In the category of OS, the primary pathway enriched was the synthesis and secretion of cortisol and aldosterone. Additionally, the pathways enriched in EIP primarily mainly encompassed the cAMP, Ras, and Hedgehog signaling pathways (Supplementary Figure S2). The KEGG enrichment analysis highlighted the top 20 pathways, predominantly emphasizing metabolism pathways such as arachidonic acid metabolism, retinol metabolism, and linoleic acid metabolism, along with pathways related to the synthesis of steroid hormones such as cortisol and aldosterone and steroid hormone biosynthesis (Figure 4B). These results indicate notable differences in metabolism, steroid synthesis, and cAMP signaling pathways between UFP and ULP, which may be closely related to the types and levels of hormones the uterus receives, as well as changes in uterine function before and after luteal formation.

### 3.3. Comparative Analysis of Gene Expression Patterns in Yak Uterus During the Follicular Phase (UFP) and Pregnant Phase (UPP)

**Figure 5:**
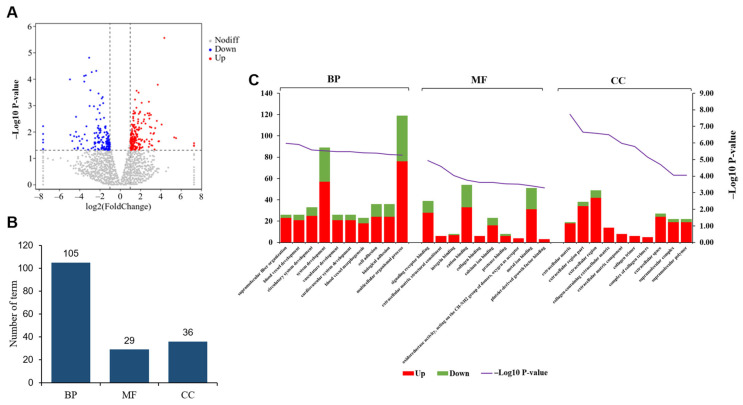
(**A**) Volcano plot displaying DEGs between yak uterus during the follicular phase (UFP) and pregnant phase (UPP). (**B**) GO terms enriched in BPs, MFs, and CCs. (**C**) GO classification chart for DEGs.

We displayed the DEGs between UFP and UPP using a volcano plot (Figure 5A). The GO analysis results revealed enrichment of the DEGs in a total of 170 significant terms (*p* < 0.01), with 105 terms enriched in BP, 29 in MF, and 36 in CC (Figure 5B and Supplementary Table S5). Similarly, based on *p*-value sorting, the top three enriched terms in BP were supramolecular fiber organization, blood vessel development, and circulatory system development; the top three enriched terms in MF were signaling receptor binding, extracellular matrix structural constituent, and integrin binding; the top three enriched terms in CC were extracellular matrix, extracellular region part, and extracellular region. Notably, in the top 10 GO terms significantly enriched in BP, MF, and CC, the number of upregulated genes exceeded the number of downregulated genes (Figure 5C).

**Figure 6:**
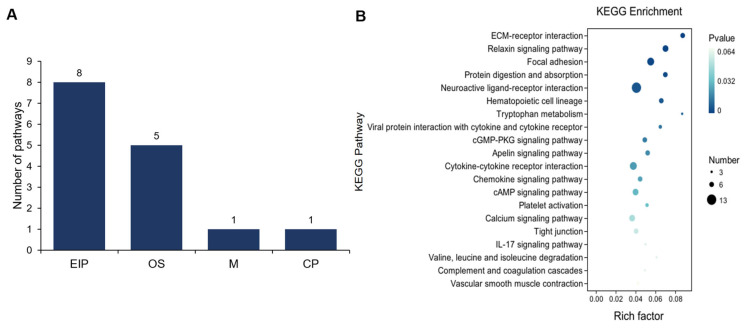
(**A**) Functional classification distribution of KEGG pathway enrichment analysis between yak uterus during the follicular phase (UFP) and pregnant phase (UPP). The functional classifications include EIP, OS, M, and cellular processes (CPs). (**B**) Bubble chart of the TOP 20 KEGG pathways.

We performed KEGG pathway enrichment analysis of the DEGs between UFP and UPP, resulting in a total of 15 significant pathways (*p* < 0.05), encompassing 8 pathways enriched in environmental information processing (EIP), 5 in organismal systems (OS), 1 in metabolism (M), and 1 in cellular processes (CPs) (Figure 6A). Notably, the pathways enriched in EIP predominantly included ECM-receptor interaction, neuroactive ligand–receptor interaction, and viral protein interaction with cytokine and cytokine receptors. The main pathways enriched in OS were the relaxin signaling pathway, protein digestion and absorption, and hematopoietic cell lineage. Finally, tryptophan metabolism and focal adhesion were enriched in CP and M, respectively. Notably, except for tryptophan metabolism, which exhibited inhibition (downregulated) in UPP, all other pathways were activated (upregulated) in UPP (Supplementary Figure S3). The KEGG enrichment results revealed that the top 20 pathways primarily involve signal transduction and signaling molecules and interactions, including ECM-receptor interaction, neuroactive ligand–receptor interaction, viral protein interaction with cytokine and cytokine receptors, and cGMP-PKG signaling pathway (Figure 6B). The results indicate substantial variations in signaling processes between UFP and UPP, potentially associated with alterations in uterine signaling mechanisms pre-and post-luteal phase. These changes may reflect modifications in endometrial structure, hormone secretion profiles, and intercellular chemical signaling involving growth factors and cytokines.

### 3.4. Comparative Analysis of Gene Expression Patterns in Yak Uterus During the Luteal Phase (ULP)and Pregnant Phase (UPP)

**Figure 7:**
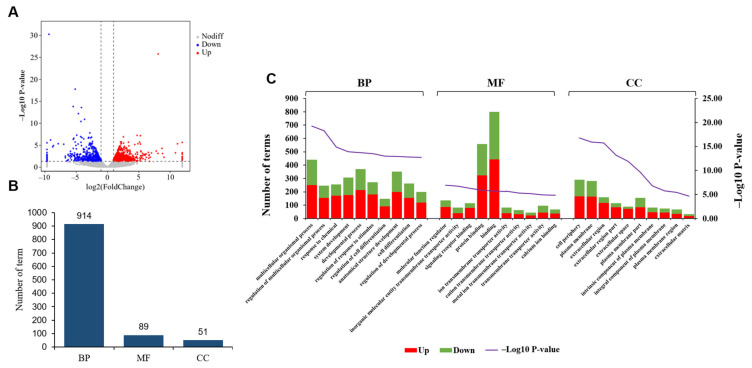
(**A**) Volcano plot displaying the DEGs between yak uterus during the luteal phase (ULP) and pregnant phase (UPP). (**B**) GO terms enriched in BPs, MFs, and CCs. (**C**) GO classification chart of DEGs.

We displayed the DEGs between ULP and UPP using a volcano plot (Figure 7A). GO analysis results indicated that DEGs were enriched in a total of 1054 significant terms (*p* < 0.01), with 914 terms enriched in BP, 89 in MF, and 51 in CC (Figure 7B and Supplementary Table S6). Similarly, the top three terms enriched in BP were multicellular organismal process, the regulation of multicellular organismal process, and the response to chemicals. The top three terms enriched in MF were molecular function regulator, inorganic molecular entity transmembrane transporter activity, and signaling receptor binding. Additionally, the top three terms enriched in CC were cell periphery, plasma membrane, and extracellular region. In the top 10 GO terms of the three categories, the number of upregulated genes is greater than the number of downregulated genes in most cases (Figure 7C).

**Figure 8:**
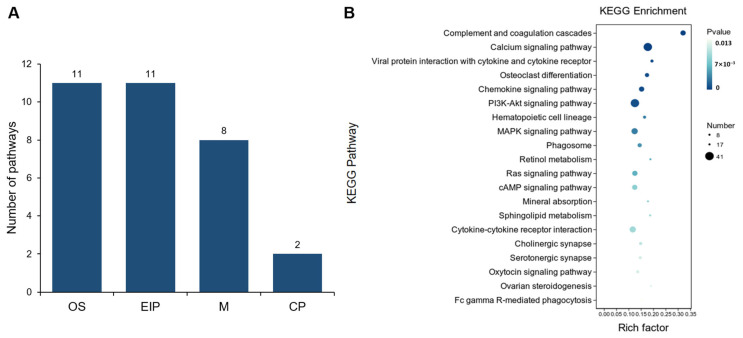
(**A**) Functional classification distribution of KEGG pathway enrichment analysis between yak uterus during the luteal phase (ULP) and pregnant phase (UPP). The functional classifications include OS, EIP, M, and CPs. (**B**) Bubble chart of the top 20 KEGG pathways.

We conducted KEGG pathway enrichment analysis of the DEGs between ULP and UPP groups, identifying a total of 32 significant pathways (*p* < 0.05), including 11 pathways enriched in OS, 11 in EIP, 8 in M, and 2 in CP (Figure 8A). Notably, the pathways enriched in OS mainly included complement and coagulation cascades, osteoclast differentiation, and the chemokine signaling pathway. In EIP, enriched pathways encompassed the calcium signaling pathway, viral protein interaction, PI3K-Akt signaling pathway, and MAPK signaling pathway. In M, pathways such as retinol metabolism, sphingolipid metabolism, and steroid hormone biosynthesis were identified. Lastly, phagosome and focal adhesion were enriched in CP (Supplementary Figure S4). The KEGG enrichment results revealed that the top 20 pathways mainly involve signal transduction pathways, cell differentiation, metabolism, and autophagy (Figure 8B), indicating a significant difference in ULP and UPP in these processes, potentially reflecting morphological and functional alterations in the yak uterus before and after pregnancy.

## 4. Discussion

The yak uterus undergoes complex physiological regulation during the follicular phase, luteal phase, and pregnant phase. These factors collectively influence the successful delivery of the fetus and impact the reproductive capacity of yaks. Upon pairwise comparison of DEGs, the ULP vs. UPP group exhibited the highest number of DEGs (1,303), significantly surpassing the counts observed in the UFP vs. ULP (495) and UFP vs. UPP (353) comparisons. Therefore, it can be concluded that at the transcriptomic level, there are more similarities in gene expression between the UFP and ULP, while greater differences in physiological regulation exist between the UFP/ULP and UPP.

In the comparison between UFP and ULP groups, the GO enrichment analysis results (Supplementary Table S4) revealed that the two most significantly enriched GO terms were biological adhesion (GO:0022610) and cell adhesion (GO:0007155), with the majority of DEGs exhibiting upregulation. Previous studies have indicated that during the menstrual cycle in mammals, the cell adhesion molecule family participates in the dynamic changes of the endometrium [25,26]. During the luteal phase, the endometrium undergoes changes to create a favorable environment for embryo attachment. The increase in adhesion molecules and the decrease in anti-adhesion molecules on the endometrial surface promote uterine receptivity, facilitating embryo implantation [27]. Our findings suggest that the transition from the follicular to luteal phase in yak uteri triggers marked activation of adhesion-related pathways, likely orchestrated by the dynamic interplay of estradiol (E2) and progesterone (P4). This steroid hormone-mediated enhancement of intercellular adhesion may constitute a critical preparatory mechanism for successful embryo attachment in the luteal endometrium.

KEGG enrichment analysis identified 14 out of the 25 significantly enriched pathways related to metabolic processes (Supplementary Table S7). Particularly noteworthy were pathways such as arachidonic acid metabolism, retinol metabolism, and steroid hormone biosynthesis, which were prominently enriched. These findings offer molecular insights into the intricate hormonal regulation governing the follicular–luteal transition in ruminants. During estrus in ruminants, ovarian follicles undergo crucial stages of recruitment, selection, and dominance, accompanied by enhanced metabolism and hormone secretion [28]. Following complete corpus luteum (CL) formation, ovarian-derived hormones—particularly estrogen and progesterone—orchestrate uterine remodeling to establish receptivity for potential embryo implantation [29]. In the absence of timely implantation, luteolysis is triggered via uterine-derived prostaglandins through a negative feedback mechanism targeting the CL [30]. Our data demonstrated significant activation of the arachidonic acid metabolism pathway in the uterine luteal phase (ULP) compared to the follicular phase (UFP). As the principal precursor of prostaglandins, arachidonic acid is stored in membrane phospholipids and mobilized for bioactive mediator synthesis [31]. Among eight DEGs annotated in this pathway, seven genes (including *EPHX2*, *GPX7*, and *PLA2G3*) exhibited marked upregulation in the ULP (Supplementary Table S7 and Table S10). *EPHX2*, a gene implicated in oxidative stress responses [32], shows elevated expression in metabolic disorders such as obesity at both transcriptional and translational levels [33]. Similarly, *GPX7* is recognized for its dual role in suppressing polyunsaturated fatty acid-induced apoptosis and scavenging reactive oxygen species (ROS) [34,35]. Although the exact regulatory mechanisms remain unclear, these findings collectively imply that *EPHX2* and *GPX7* may contribute to uterine metabolic adaptation during luteal phase progression in ruminants. Specifically, their upregulated expression in yak uteri could facilitate endometrial preparedness for embryo implantation. *PLA2G3*, on the other hand, can promote the maturation and mediator secretion of mast cells [36]. Research has indicated a notably higher distribution of mast cells in the cervix of buffaloes during the luteal phase compared to the follicular phase [37], aligning with the expression pattern of *PLA2G3* in the yak uterus. We hypothesize that the upregulation of the *PLA2G3* gene in the yak uterus from the follicular phase to the luteal phase is associated with an increase in mast cells, potentially facilitating their accelerated maturation. Nonetheless, this conclusion requires further validation. Additionally, compared to the UFP, the expression level of the *AdipoR2* gene is significantly increased during the ULP. *AdipoR2* has been identified in the human endometrium [38], and reports have indicated that the mRNA expression levels detected in the mid-luteal phase of the pig hypothalamus are significantly increased [39]. This finding is consistent with our research results (Supplementary Table S10), collectively indicating that *AdipoR2* may play a role in the uterine preparation for potential implantation.

In the ULP vs. UPP group, the results of the GO enrichment analysis (Supplementary Table S6) highlighted the multicellular organismal process (GO:0032501) and regulation of multicellular organismal process (GO:0051239) as the two most significantly enriched GO terms, with a relatively large number of both upregulated and downregulated genes. We speculate that the significant enrichment of GO terms related to multicellular organism processes is primarily due to the complex physiological regulation during the two stages and the cellular-level changes that occur. During the luteal phase, the uterus needs to provide a suitable microenvironment for embryo development. The GO terms related to multicellular organism processes may reflect cellular proliferation, differentiation, and tissue remodeling in the uterus at this stage, encompassing alterations in various cell types (e.g., epithelial cells, smooth muscle cells, and endothelial cells). During the first trimester of pregnancy, the uterus undertakes intricate processes such as decidualization, placental formation, and vascular remodeling, all of which rely on the coordinated efforts of various cell types, including endometrial stromal cells, immune cells, and endothelial cells. In the KEGG enrichment analysis, the *IGF1* gene was notably enriched in the MAPK and PI3K/Akt signaling pathways (Supplementary Table S8), with FPKM values exceeding 100 in all three phases, peaking during the pregnancy phase (Supplementary Table S10). Reports have indicated that *IGF1* can activate the MAPK and PI3K/Akt signaling pathways [40,41]. The anti-apoptotic function of *IGF1* requires the PI3K signaling pathway, while its proliferative effects depend on the MAPK signaling pathway, which is consistent with our research findings. We speculate that the higher expression of the *IGF1* gene during pregnancy indicates its role in promoting uterine development and fetal growth by inhibiting apoptosis and promoting cell proliferation.

Furthermore, the differential expression analysis of ULP vs. UPP identified IHH as the most significantly downregulated gene (Supplementary Table S3). KEGG enrichment analysis demonstrated the exclusive involvement of this gene in the Hedgehog signaling pathway, where six of eight pathway-associated DEGs (including *IHH*, *GLI1*, *PTCH1*, *PTCH2*, *LRP2*, and *SMO*) were downregulated (Supplementary Table S8). Similarly, in terms of the KEGG results for UFP vs. ULP, the Hedgehog signaling pathway remains significantly enriched, with six DEGs enriched, four of which are upregulated. The upregulated genes included *IHH*, *GLI1*, *PTCH1*, and *PTCH2* (Supplementary Table S7). The Hedgehog (HH) signaling pathway, first identified in Drosophila in 1980, was evolutionarily conserved across species [42]. According to previous studies [43], among the HH signaling molecules in mammals, Indian hedgehog (IHH) is one of the three ligands, while *PTCH1* and *PTCH2* act as the two PTCH membrane receptors; *SMO* functions as the sole signal transducer in this pathway, and *GLI1* act as a transcription factor associated with glioma oncogenes. The expression levels of the aforementioned genes associated with the HH signaling molecules (except for *SMO*) are significantly higher during the luteal phase compared to the follicular and luteal phases. Studies have shown that the expression of *IHH* and *GLI1* genes in the human endometrium is notably elevated during the secretory phase compared to the proliferative phase [44], aligning with our research outcomes. These results suggest a close association between the Hedgehog signaling pathway and the luteal phase-specific changes in the uterus. However, investigations into the Hedgehog signaling pathway within the uterus have predominantly centered on humans and mice to date. Further exploration is warranted to delineate the specific interplay between Hedgehog signaling and the yak luteal-phase uterus.

In the comparison between UFP and UPP groups, the Gene Ontology (GO) enrichment analysis demonstrated (Supplementary Table S5) that the two most significantly enriched GO terms were extracellular matrix (GO:0031012) and extracellular region part (GO:0044421), with the majority of enriched genes exhibiting upregulation patterns. We speculate that this phenomenon is related to the extensive tissue remodeling required in the uterus during the third month of pregnancy. At this stage, maternal blood vessels provide sufficient nutrients and oxygen to the fetus, with the uterine vascular density and blood flow velocity significantly exceeding those during the follicular phase. The observed upregulation of extracellular matrix (ECM)-related genes likely represents an active regulatory mechanism to support uterine expansion for fetal growth and pregnancy maintenance. The KEGG enrichment analysis results indicate that DEGs are significantly enriched in signaling pathways such as ECM-receptor interaction, relaxin signaling pathway, focal adhesion, and protein digestion and absorption (Supplementary Table S9). The interactions between ECM receptors and focal adhesions are important pathways involved in activation changes [45]. ECM is a complex network structure comprising diverse proteins and non-proteins [46], serving not only as attachment sites and structural support for cells but also as a source of vital biological information that regulates cellular activities and organ functions [47]. Research reports have highlighted the presence of various ECM proteins on the surface of uterine stromal cells [48,49], where these cell surface glycoproteins contribute to epithelial–embryo interactions. Investigations have demonstrated substantial ECM remodeling in the uterine endometrial stroma of mice during early pregnancy, playing a critical role in embryo implantation and development [50]. In our analysis, we found that ECM-receptor interaction was the most significantly enriched pathway, with seven upregulated DEGs enriched in this pathway. Additionally, the focal adhesion pathway ranked within the top three for enrichment, featuring 10 upregulated DEGs linked to this pathway (Supplementary Table S9). Both pathways share six common upregulated genes, including FN1, COL1A2, ITGA5, COL4A2, and COL4A1, displaying heightened expression levels in UPP. FN1 has an FPKM value greater than 100 from the follicular phase to the pregnancy phase, with a significant increase in its expression level (Supplementary Table S10). Fibronectin, encoded by the FN1 gene, is a major component of the ECM, typically existing as dimers or multimers on the cell surface and in the ECM. It plays a vital role in essential biological processes such as cell migration, adhesion, and tissue repair [51]. FN1 (Fibronectin 1) plays a crucial role in the adhesion and implantation of the blastocyst into the uterus [52] and is essential in mammalian reproduction and placental development [53]. Therefore, we predict that during the luteal phase, the FN1 gene may prepare the yak uterus for embryo implantation by regulating ECM protein synthesis. At three months of pregnancy, FN1’s involvement may be linked to material exchange between the uterus and fetus through the placenta during this stage. FN1 likely promotes cell differentiation and angiogenesis, contributing to the maintenance of maternal pregnancy.

Interestingly, in the UFP vs. UPP group, the DEGs significantly enriched in the top four signaling pathways included *COL1A2*, *COL4A1*, and *COL4A2*. The *COL3A1* gene is involved in three significantly enriched signaling pathways (the relaxin signaling pathway, protein digestion and absorption, and platelet activation). These genes belong to the collagen family, encompassing various collagen types such as type I, II, III, and IV. These collagens are important components of the ECM that maintain its structure and function. Type I collagen consists of an α1 chain encoded by the *COL1A1* gene and an α2 chain encoded by the *COL1A2* gene [54]. *COL3A1* encodes type III collagen, a major component of hollow organ structures like the uterus, while *COL4A1* and *COL4A2* genes encode the most common type IV collagen in the body [55], serving as a fundamental structural component of the basement membrane. Our results indicate that the expression levels of *COL1A1*, *COL3A1*, *COL4A1*, and *COL4A2* are relatively high in all three periods (with FPKM values exceeding 200), and their expression gradually increases from the follicular phase to the pregnancy phase, peaking during pregnancy (Supplementary Table S10). Collagen, a major component of connective tissue, participates in the synthesis and repair of the ECM and is essential for maintaining tissue morphology and mechanical properties. Based on its functional relevance to tissue remodeling, we hypothesize that as the yak uterus transitions from the follicular phase to pregnancy, there is a gradual upregulation in the expression of specific collagen family genes. This upregulation likely contributes to the accumulation of ECM components (e.g., fibronectin and elastin), thereby facilitating uterine remodeling, fetal growth, and pregnancy maintenance. These dynamic alterations in collagen expression are essential for ensuring that the uterus adapts effectively to the diverse physiological requirements across different reproductive stages.

In summary, the physiological regulation of the yak uterus during the estrous cycle and pregnancy is complex. During the follicular phase, estrogen and progesterone secreted by the ovaries primarily govern the structural changes in the yak uterus. In the luteal phase, the formation of the corpus luteum leads to the secretion of large amounts of progesterone, causing the uterine lining to thicken and increasing the number of blood vessels, thereby providing sufficient nutrients and oxygen for embryo implantation and growth. With the onset of pregnancy, the corpus luteum secretes progesterone to maintain pregnancy. As the placenta develops, it assumes the role of the primary site for progesterone production. The fetus establishes a connection with the uterus through the placenta, enabling the transfer of various growth factors and enhanced gene expression from the mother to support optimal fetal growth and development. From the follicular phase to pregnancy, the yak uterus continually prepares for material demands, with the number of blood vessels within the uterus gradually increasing. The DEGs enriched in signaling pathways are closely associated with hormone regulation, physiological structural changes of the uterus, and placental formation and function, as well as fetal growth and development.

## 5. Conclusions

This study included a transcriptomic analysis and pairwise comparative analyses among the follicular, luteal, and pregnant phases to identify the key genes and pathways regulating the dynamic changes of the uterus across the yak reproductive cycle. In the UFP vs. ULP group, significantly enriched KEGG pathways were primarily related to lipid metabolism, hormone synthesis, and secretion, indicating that the differences between the two phases are primarily driven by hormonal fluctuations. Genes such as *EPHX2* and *GPX7* are implicated in the preparatory processes of the uterus preceding pregnancy. In the UFP vs. UPP group, the ECM-receptor interaction pathway was the most significantly enriched. Specifically, collagen genes associated with ECM synthesis, including COL1A2, COL3A1, COL4A1, and COL4A2, were markedly upregulated in UPP. This upregulation may suggest enhanced deposition of ECM components (e.g., fibrillar collagens and glycoproteins) during pregnancy. Such enhanced ECM deposition likely contributes to uterine tissue remodeling, establishing a mechanically stable microenvironment conducive to support fetal growth and placental development in yak. In the ULP vs. UPP group, the significantly enriched pathways encompass the complement and coagulation cascades, calcium signaling pathway, and osteoclast differentiation signaling pathway. These pathways are involved in hormone secretion, cell differentiation, uterine myometrium regulation, and immune mechanism modulation. Their interactions collectively maintain the normal function of the uterus and the stability of pregnancy. This study investigated the functional regulation of DEGs in the yak uterus during different reproductive stages in physiological processes such as hormone secretion, uterine development, and fetal growth and development, providing a foundational framework for enhancing yak reproductive efficiency.

## Figures and Tables

**Table 1:** Primers used in the present study.

Gene Name	Sequence	Lengths (bp)
*GSN*	F: CACTACTGGCTGGGCAATGA R: CCGTTCAGGTAGTCGTCCAG	86
*VIM*	F: CCCTGAACCTGAGGGAAACC R: CGTGATGCTGGGAAGTTTCG	129
*FOS*	F: CGTCAATGCGCAGGACTACT R: GGAGACTAGGGTGGGCTGTA	121
*GLI1*	F: CGCCCAGACAGAGTGCC R: GCGGATAACTGTCTGCAGGT	120
*β-ACTIN*	F: GCAATGAGCGGTTCC R: CCGTGTTGGCGTAGAG	141

F: forward primer; R: reversed primer.

## Data Availability

Data are contained within the article Supplementary Materials.

## Supplementary Materials

The following supporting information can be downloaded at https://www.mdpi.com/article/10.3390/ani15060837/s1. Supplementary Figure S1: Clustering heatmap of differentially expressed genes (DEGs) based on FPKM values. Supplementary Figure S2: Top 5 KEGG pathways at the first classification level in ULP vs. ULP (*p*-value < 0.05). Supplementary Figure S3: Top 5 KEGG pathways at the first classification level in ULP vs. UPP (*p*-value < 0.05). Supplementary Figure S4: Top 5 KEGG pathways at the first classification level in ULP vs. UPP (*p*-value < 0.05). Supplementary Figure S5: raw data results (post-sequencing data) and gene coverage uniformity. Supplementary Table S1: UFP vs. ULP DEGs. Supplementary Table S2: UFP vs. UPP DEGs. Supplementary Table S3: ULP vs. UPP DEGs. Supplementary Table S4: UFP vs. ULP. significant GO enrichment (*p*-value < 0.01). Supplementary Table S5: UFP vs. UPP. significant GO enrichment (*p*-value < 0.01). Supplementary Table S6: ULP vs. UPP. significant GO enrichment (*p*-value < 0.01). Supplementary Table S7: significantly enriched ULP vs. UFP. KEGG signaling pathways (*p*-value < 0.05). Supplementary Table S8: significantly enriched ULP vs. UPP. KEGG signaling pathways (*p*-value < 0.05). Supplementary Table S9: UFP vs. UPP. KEGG significantly enriched signaling pathways (*p*-value < 0.05). Supplementary Table S10: The FPKM values of some differentially expressed genes (DEGs) mentioned in the article (*p*-value < 0.05).
