# Supplementary material for: Transcriptomics Reveals the Differences in mRNA Expression Patterns in Yak Uterus of Follicular, Luteal, and Pregnant Phases
Source: Animals (Basel). 2025 Mar 14;15(6):837. doi: 10.3390/ani15060837 (PMC11939727; doi:10.3390/ani15060837)
Supplement: Supplementary file 1 [file animals-15-00837-s001.zip › animals-3455919-supplementary/Supplementary Figure S1.pdf]

Supplementary Figure S1: Clustering Heatmap of Differentially Expressed Genes (DEGs) Based on FPKM Values

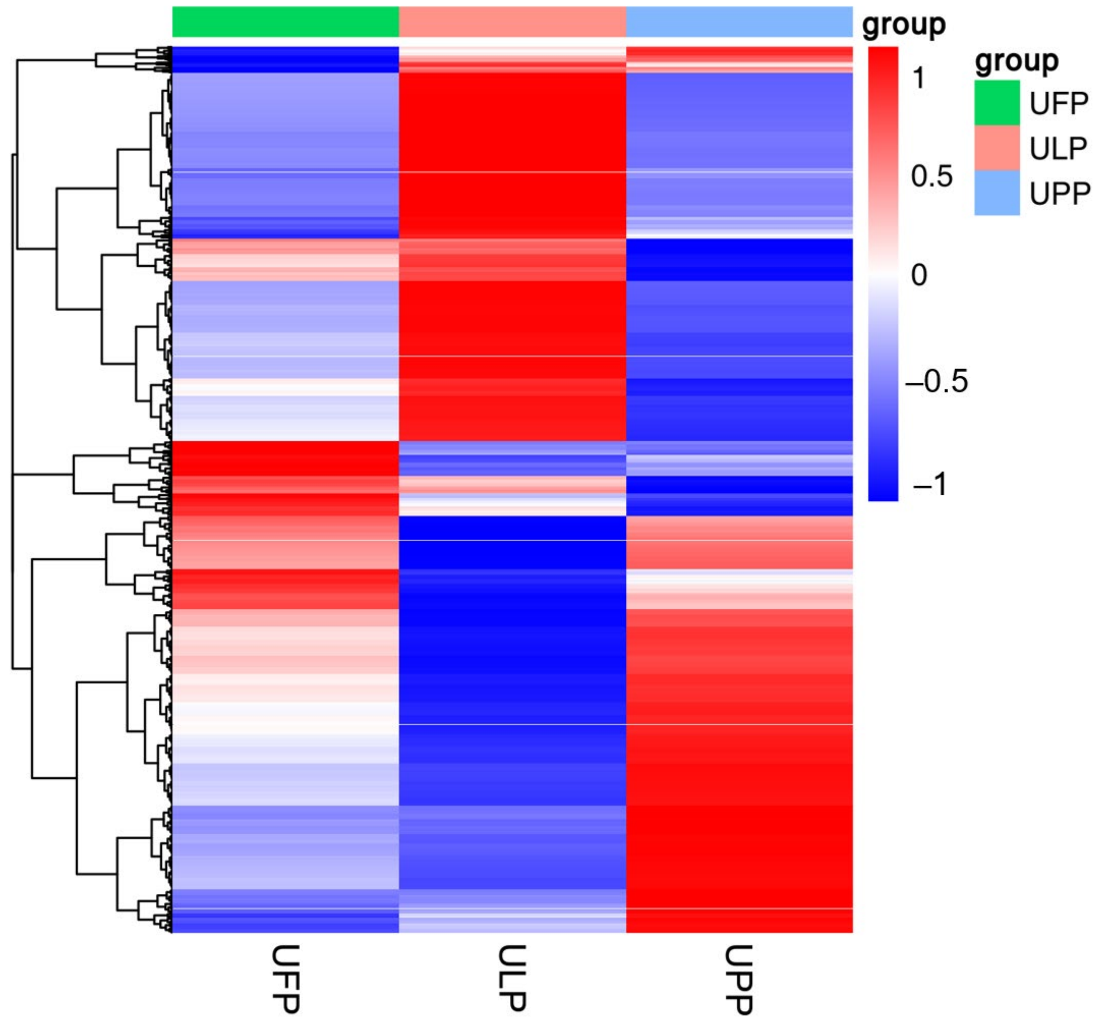

Figure legend: Genes are represented horizontally, with each column corresponding to a group. Red indicates high expression genes, and green indicates low expression genes.
