# Supplementary material for: Transcriptomics Reveals the Differences in mRNA Expression Patterns in Yak Uterus of Follicular, Luteal, and Pregnant Phases
Source: Animals (Basel). 2025 Mar 14;15(6):837. doi: 10.3390/ani15060837 (PMC11939727; doi:10.3390/ani15060837)
Supplement: Supplementary file 1 [file animals-15-00837-s001.zip › animals-3455919-supplementary/Supplementary Figure S3.pdf]

Supplementary Figure S3: Top5 KEGG pathways at the first classification level in UFP vs UPP

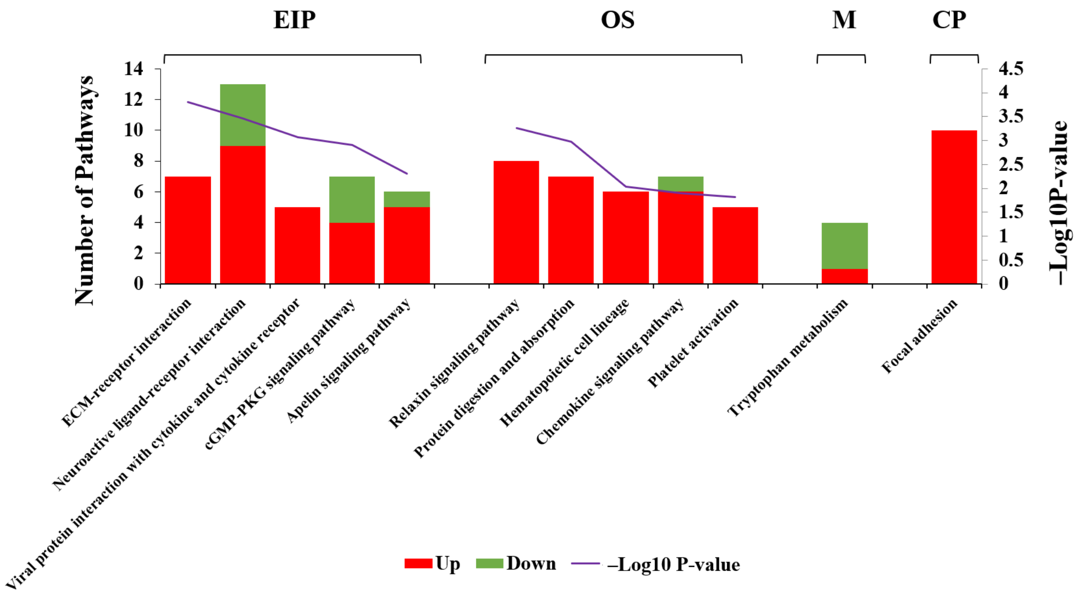

Figure legend: The x-axis represents the significantly enriched signaling pathways. The left y-axis corresponds to the bar chart, representing the number of differentially expressed genes enriched in each signaling pathway. Red bars indicate up regulated genes, and green bars indicate down regulated genes. The right y-axis corresponds to the line chart, representing the –Log10 P-value.

Figure note:

M: Metabolism;

OS: Organismal Systems

EIP: Environmental Information Processing;

GIP: Genetic Information Processing
