# Supplementary material for: Transcriptomics Reveals the Differences in mRNA Expression Patterns in Yak Uterus of Follicular, Luteal, and Pregnant Phases
Source: Animals (Basel). 2025 Mar 14;15(6):837. doi: 10.3390/ani15060837 (PMC11939727; doi:10.3390/ani15060837)
Supplement: Supplementary file 1 [file animals-15-00837-s001.zip › animals-3455919-supplementary/Supplementary Figure S5.pdf]

Supplementary Figure S5: Raw Data results (Post-sequencing data) and Gene Coverage Uniformity

Table title: Raw Data results (Post-sequencing data)

Sample	Raw Read Number	Raw Bases	Raw Q30 number	Raw N rate	Raw_Q20_rate	Raw_Q30_rate
UFP1	43484172	6566109972	6236241500	0.00028	98.27	94.97
UFP2	53586888	8091620088	7636944021	0.000268	98.04	94.38
UFP3	50729886	7660212786	7228265457	0.000274	98.03	94.36
ULP1	46627072	7040687872	6638855668	0.000268	97.99	94.29
ULP2	42185758	6370049458	6008700185	0.000273	98.01	94.32
ULP3	44911630	6781656130	6390429629	0.000281	97.98	94.23
UPP1	49802392	7520161192	7125501427	0.000279	98.19	94.75
UPP2	40328340	6089579340	5739746210	0.000269	97.98	94.25
UPP3	47986036	7245891436	6844773566	0.061306	98.03	94.46

Sample: Sample name

Reads Number: Total number of reads

Bases (bp): Total number of bases

Q30 (bp): Total number of bases with base call accuracy >99.9%

N (%): Percentage of ambiguous bases

Q20 (%): Percentage of bases with base call accuracy >99%

Q30 (%): Percentage of bases with base call accuracy >99.9%

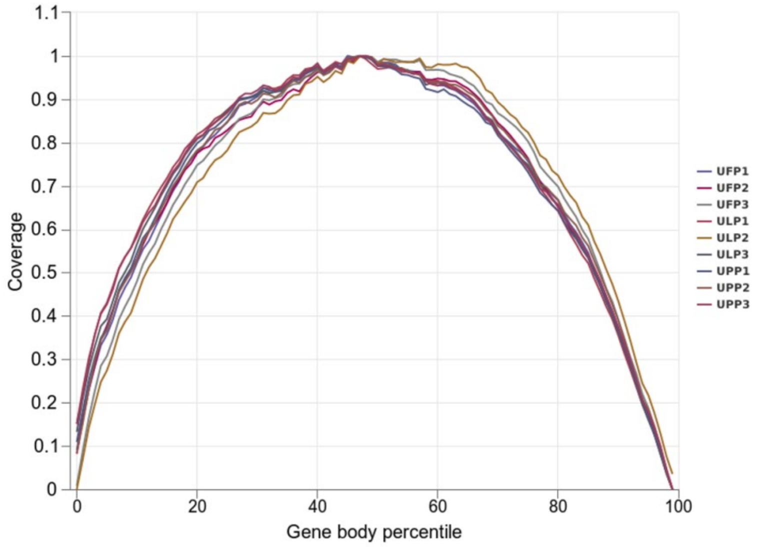

Figure title: Gene Coverage Uniformity

Figure legend: The horizontal axis represents the percentage of a single gene's base length relative to its total length, with 0 indicating the 5' end of the gene and 100 indicating the 3' end. The vertical axis represents the total number of sequences aligned to the corresponding intervals across the horizontal axis for all genes.
